# ‘Today I am so happy to see friends I once worked with many years ago’

**DOI:** 10.1098/rstb.2008.4015

**Published:** 2008-11-27

**Authors:** Inamba Kivita

**Affiliations:** Wanitabi Village, c/o Papua New Guinea Institute of Medical ResearchPO Box 60, Goroka, EHP 441, Papua New Guinea

I was approximately 16 years old when I first met Dr Carleton Gajdusek in Wanitabi village, situated south of Okapa in the Eastern Highlands Province. I worked with Shirley Lindenbaum when she came to live in our village, and I helped Michael Alpers with his research. I was asked to work as a translator, also to assist with the fieldwork and carry personal things like camera, books and film. The older men carried the heavy boxes from one village to another on kuru surveillance patrols.

There were other medical scientific officers who came later with whom I worked as well, such as Dr Hornabrook and John Mathews. I was trained by them to perform autopsies on kuru dead bodies. Though my position with the project was as a translator, sometimes it was my duty to take human samples collected from the field to Goroka by plane from Tarabo airstrip and return back to the field by the same route.

One of the colleagues who helped me was Tosetnam from Miarasa village; we both shared the workload and helped in the fieldwork. Some of our comrades are not here owing to medical reasons and some, like Tosetnam, have already died. Today I am so happy to see friends I once worked with many years ago, in the 1960s and 1970s.

